# Development of *Phaseolus vulgaris *endogenous reference and Embrapa 5.1 event-specific assays for quantification of Embrapa 5.1 GM common bean using real time PCR

**DOI:** 10.1186/1753-6561-8-S4-P116

**Published:** 2014-10-01

**Authors:** Diana Treml, Gustavo Venturelli, Fabio Brod, Ana Carolina Arisi Maisonnave

**Affiliations:** 1 Federal University of Santa Catarina, Florianópolis, Brazil

## Background

Brazil is the second largest producer of genetically modified (GM) crops with 37 GM crop varieties approved by CTNBio for commercialization. One of these varieties is the Embrapa 5.1 common bean, resistant to the bean golden mosaic virus (BGMV). The Embrapa 5.1 event was developed using the RNAi concept to induce the silencing of the AC1 viral gene [1]. Labelling of food products containing GMOs in Brazil is mandatory [2], so methods for quantification of GMOs are required. Real time PCR methods have been used as the gold standard for GMO quantification. In a previous work, we have developed a construct-specific PCR detection assay for Embrapa 5.1 [3]. In this study we developed an event-specific PCR assay for Embrapa 5.1.

## Methods

Genomic DNA was extracted from leaves using two different protocols, a CTAB method and the DNeasy Plant Mini Kit with modifications [3].

Quantification of the endogenous reference was performed using primers and Taqman probe targeting the lectin gene present in common bean (*Phaseolus vulgaris*) genome. The reaction parameters efficiency and limit of detection (LOD) were determined using conventional and GM varieties Perola and Pontal by 10-fold serial dilution of the genomic DNA ranging from 10^5 ^to 10^0 ^genome copies. The assay specificity was performed using 50 accessions of *Phaseolus vulgaris *and 13 different crop species including maize, GM maize varieties, soybean and GM soybean RR.

Quantification of the event-specific fragment was performed using primers and probe targeting the event-specific junction of the Embrapa 5.1 event. The reaction parameters efficiency and limit of detection (LOD) were determined for the variety Perola GM by 10-fold serial dilution of the genomic DNA ranging from 10^5 ^to 10^0 ^genome copies. The specificity assay was performed using the Embrapa 3.2 event, also resistant to the BGMV. Primers and probe concentrations were tested in order to determine the more suitable reaction efficiency.

## Results and conclusions

The endogenous reference presented an efficiency of 96% and a LOD of 10 genome copies. This target was specific for the quantification of *Phaseolus vulgaris *varieties and no amplification was observed in 10 out of thirteen negative controls. The remaining three negative controls presented late Cts (Ct>34). Also, this assay presented similar Cts among the fifty accessions of *Phaseolus vulgaris*.

The event-specific detection presented efficiencies ranging from 104 to 111%. A LOD of 1 genome copy was obtained. Also, this assay was specific for the Embrapa 5.1, although four out of twelve reactions were positive for the Embrapa 3.2 event. Even so, these four amplification presented late Cts (Ct>38). The primers and probes developed in this work are suitable for Taqman real time PCR quantification of Embrapa 5.1.
